# Geriatric Assessment Tools in Head and Neck Radiation Oncology: An Unmet Need

**DOI:** 10.7759/cureus.79979

**Published:** 2025-03-03

**Authors:** Rebecca E McIver, Lily Ottensoser, Bhupesh Parashar

**Affiliations:** 1 Radiation Oncology, Zucker School of Medicine at Hofstra/Northwell, Hempstead, USA

**Keywords:** assessment, geriatrics, oncology, radiation, toxicity

## Abstract

The geriatric population faces unique challenges in cancer treatment due to higher rates of comorbidities, which can complicate the risk-benefit analysis of treatment regimens and overall treatment decisions for both physicians and patients. This is especially true for head and neck cancers (HNC) since these patients experience significant treatment-related morbidity. Currently, there are several geriatric assessment (GA) tools available to predict outcomes in older cancer patients treated with surgery or chemotherapy, but no such tool exists to assess the frailty of geriatric patients undergoing radiation therapy for HNC. In this review, we discuss the available geriatric tools, especially those meant for cancer patients, their limitations in HNC patients, and an additional limitation of predicting radiation (RT) treatment outcomes in this challenging group of patients. We also present preliminary data for a new GA tool for HNC patients that can predict premature termination (PT) of treatment or extended treatment (ET) time.

## Introduction and background

Challenges in geriatric oncology assessment in head and neck cancers (HNC) 

With demographic changes in the United States, there is a growing aging population, and an increasing number of patients over 65 years are being diagnosed and treated for various types of cancer. Fifty-eight percent of the new invasive cancers reported in the United States were in adults over the age of 65 years [1]. Adults over 85 years of age made up 2% of the United States population but 8% of the new cancer diagnoses in 2019 [2]. The geriatric population faces unique challenges in cancer treatment with higher rates of comorbidities, which can complicate treatment decision-making and can make weighing the risks and benefits of certain treatment regimens a challenge for both physicians and patients. This is especially true for HNC that are associated with significant treatment-related morbidity [3]. A recent large systematic review found that 42% of older cancer patients over 70 years of age were considered frail, and frailty was associated with an increased risk of postoperative mortality, intolerance to cancer treatment, and post-operative complications [4]. These data highlight the increasing need for multidisciplinary teams that treat cancer patients to be familiar with the unique challenges that geriatric cancer patients face and for new tools to be developed to detect areas of risk more successfully. 

The gold standard for geriatric assessment (GA) is the comprehensive GA (CGA), although not specific for HNC, which has been shown to be better at predicting postoperative outcomes than chronological age [5]. Despite its accuracy, it is time-consuming and often requires input from several members of a multidisciplinary team. Some general GA tools such as the Eastern Cooperative Oncology Group (ECOG) Performance Status (PS) have been used as a substitute for more intensive screening but can fail to capture the nuances of risk factors and frailty in the geriatric cancer population. Additional screening tools, such as the Vulnerable Elders Survey-13 (VES-13), Geriatric-8 (G8), and the Senior Adult Oncology Program-2 (SAOP2), have been developed to assess frailty in the geriatric population. However, not all these tools are specific to oncologic patients, or HNC, and do not necessarily account for the different physical and mental demands of medical oncologic treatment vs. surgical oncologic treatment vs. predicting radiation (RT) in the HNC population. 

Head and neck RT has a significant side effect burden that can lead to decreased quality of life and premature termination (PT) or delays to completion of radiotherapy in all age groups. Some of the most common side effects include mucositis, odynophagia, dysphagia, hoarseness, xerostomia, pain, dermatitis, hair loss, nausea, and vomiting [6]. A recent study investigated the effects of age and frailty on acute radiation-induced toxicity in patients being treated for HNC and found that age >65 years was associated with increased rates of RT toxicity [7]. This study used two GA tools, the G8 and Groningen Frailty Index (GFI), to assess frailty and found that increased frailty was not associated with increased early side effect burden [7]. These findings suggest that current GA tools do not sufficiently predict the risk of increased side effects and PT of RT for HNC.

In the realm of medical oncology, tools such as the Chemotherapy Risk Assessment Scale for High-Age Patients (CRASH) and Cancer and Aging Research Group and Chemotherapy Toxicity Tool (CARG-TT) were created to determine the risk of severe chemotherapy-induced toxicity in older patients [8,9]. The electronic Rapid Fitness Assessment (eRFA) has been developed for use in the field of surgical oncology to risk stratify geriatric cancer patients preoperatively [10]. Unlike medical oncology and surgical oncology, there are no specific standardized tools for assessing risk for toxicity specifically for geriatric patients undergoing radiotherapy in the HNC population. 

Many tools have been developed to assess overall functional status in geriatric patients and risk of detrimental changes to function because of cancer treatment, but none are specific to RT. For the purposes of this review, the geriatric population will be defined as patients over 65 years. The search strategy for this review involved a PubMed search using the following terms: geriatric oncology, geriatric compliance, PT, head and neck cancer, and head and neck cancer radiation. This review seeks to summarize the current tools being used in geriatric oncology to assist with risk assessment and treatment decision-making, while highlighting the need for more specific tools to be created and utilized specifically in the field of HNC RT. 

## Review

CGA 

CGA is a broad diagnostic and treatment tool used in the geriatric population to evaluate functional limitations and help drive treatment decisions. The major areas of the assessment include ability to perform functional tasks and need for assistance, fall history, urinary and/or fecal incontinence, pain, sources of social support, depressive symptoms, vision, or hearing difficulties, and whether the patient has completed a durable power of attorney for health care [11]. The main drawback to the use of the CGA routinely in elderly cancer patients is that it is time-consuming and requires training to be able to administer. Hurria et al. found that the average time to complete a modified CGA specific to oncology was 27 minutes [12]. 

Antonio et al. performed a prospective study of 85 patients over 75 years of age with inoperable, locally advanced non-small cell lung cancer who underwent both CGA and VES-13 prior to treatment [13]. The patients who were fit or medium fit by CGA received concurrent chemoradiation (CRT), while those deemed unfit received supportive care. Patients who were deemed fit or medium fit had longer median overall survival compared to unfit patients. Patients deemed fit or medium fit by CGA but with higher VES-13 scores had a shorter median overall survival [13]. This study suggests that CGA may uncover more subtle impairments in functioning than PS, which could help guide treatment decision-making, and patients most in need of these more time-intensive assessments may be identified using tools like the VES-13. 

In a study evaluating CGA in post-operative patients with HNC, patients aged ≥70 years who underwent elective HNC surgery participated in a study that used CGA scores to determine their association with postoperative complications. Thirty-four of 65 patients (52.3%) with deficits in two or more preoperative CGA domains were categorized as “frail.” Frailty assessed by preoperative CGA, but not chronological age, was significantly associated with major postoperative complications [5]. 

Pottel et al. evaluated the diagnostic performance of CGA plus additional screening tools [VES-13, G8, and the combined screening tool ‘VES-13 + (17-G8)’ or CST] and correlations with health-related quality of life evolution (HRQOL and European Organization for Research and Treatment of Cancer Quality of Life Questionnaires (EORTC QLQ)-C30 and -HN35] during RT. In 100 patients, aged ≥65 years, with HNC undergoing curative radio(chemo)therapy, G8 was the screening tool of choice. Serial CGA identified the evolution of multidimensional health problems and HRQOL conditions during therapy with potential to guide individualized supportive care [14]. 

Another retrospective study evaluated the impact of CGA in treatment decision and outcome and compared it to a control cohort with no CGA treated within the same institution. CGA changed final treatment decision following tumor board in 31% of the cases. Patients were more likely to receive standard treatment in the CGA cohort when compared to the control (36 vs. 21%; p = 0.048), with no differences observed in treatment completion rate (84% vs. 86%; p = 0.805). In patients who underwent conservative treatment, overall response rate was similar between CGA and control cohort [15]. In addition, there have been additional studies that study the benefit of CGA in HNC to assess frailty and toxicity [16,17]. 

Despite its use for a general frailty assessment in HNC patients undergoing treatment, whether it’s surgery, RT, chemotherapy, or a combination, CGA is limited in assessing toxicity and PT of RT in this group of patients. 

ECOG PS 

ECOG PS is the most widely used tool by oncologists to make treatment-related decisions. This scoring system ranges from 0 being fully active, 1 being restricted with strenuous exercise, 2 being able to perform self-care and be up and about 50% of the time, 3 being able to carry out some self-care but confined to bed or chair more than 50% of the time, 4 being unable to carry out self-care and confined to bed or chair 100% of the time, and 5 being deceased [18]. 

Datta et al. explored the variability in ECOG rating between oncology clinicians using clinical case vignettes [19]. The study found that there was poor agreement in the ECOG ratings among different raters with concordance rates ranging from 19.4% to 56.9%. Certain factors, such as age, perceived socioeconomic background, patient preferences, and past treatment response, were shown to impact decision-making [19]. These data are alarming in that, currently, PS can have a profound impact on the types of therapy offered and overall patient outcomes in oncology, but it may lack the objectivity necessary to be sufficiently accurate and effective. More objective methods of screening elderly patients must be employed to ensure that patients are receiving appropriate care with clear expectations of the risks and benefits. 

ECOG PS has been shown to predict outcomes in HNC management as well as RT completion [20]. In a recently published phase 3 trial (Guigay et al.) to compare the efficacy and safety of cetuximab to those of methotrexate in recurrent/metastatic HNC that used ECOG PS of 0-2 as inclusion, patients were randomly assigned (1:1) to receive cetuximab 500 mg/m² intravenously every two weeks or methotrexate 40 mg/m² intravenously every week, with minimization by ECOG PS, type of disease evolution, Charlson Comorbidity Index (CCI) score, serum albumin concentration, and geriatrician consultation [21]. There was no improvement in failure-free survival with cetuximab vs. methotrexate, and patients with an ECOG PS of 2 did not benefit from these systemic therapies. 

Our institutional study showed ECOG status (Cooper et al.) to predict premature RT termination in oral cavity and laryngeal cancers [20]. However, ECOG PS alone has not been shown to predict RT toxicity and RT treatment termination in a prospective setting [20]. 

VES-13 

VES-13 was developed as a self-reported screening tool to assess the increased risk of death or functional decline in patients over 65 years, but not specifically in the oncologic population [22]. It is function-based and assesses age, self-rated health, and the need for assistance with multiple different activities of daily living (ADLs) and instrumental ADLs (IADLs), including shopping, managing money, housework, transferring, bathing, etc. A score of >3 classifies patients as vulnerable with a 4.2 times higher risk of death or functional decline over a two-year period [22]. 

Spyropoulou et al. performed a prospective observational study on 230 patients over 75 years with cancer who were referred for radical or palliative radiotherapy and compared VES-13 scores before starting treatment [23]. They found that patients who did not complete radiotherapy had higher VES-13 scores and that a score of >3 was associated with a 2.14 times higher chance of failing to complete radiotherapy, while a score of >7 was associated with a 3.34 times higher chance of failing to complete radiotherapy [23]. 

Antonio et al. also investigated the association between CGA, VES-13, and toxicity [13]. Interestingly, the fitness level as defined by the CGA did not correlate with a statistically significant increase in the rate of grade 3-4 toxicity, but patients deemed vulnerable by the VES-13 had significantly higher rates of grade 3-4 toxicity compared to non-vulnerable patients. Additionally, upon logistic regression, CGA-defined groups were not predictive of grade 3-4 toxicity outcome, but the VES-13-defined groups correlated with a significantly higher risk of grade 3-4 toxicities with an odds ratio of 3.99 [13]. These data suggest that VES-13 may be helpful in predicting the risk of moderate and severe toxicity in patients undergoing concurrent CRT. 

A retrospective study was conducted on cancer patients attending a geriatric oncology clinic between July 2015 and June 2017, who completed a VES-13 [24]. Patients were stratified into those who were “VES-13 positive” (score ≥ 3) and “VES-13 negative” (score < 3). Logistic regression was used to explore the relationship between VES-13 score and treatment modification. VES-13 score was predictive of treatment plan modification (63.0% among VES-13 positive vs. 16.7% among VES-13 negative patients; p = 0.001). The authors concluded that ‘VES-13 may provide oncologists with a rapid, reliable way of identifying vulnerability in older adults with cancer who may need further GA prior to commencing cancer treatment’. 

G8 

The G8 screening tool was developed specifically for geriatric cancer patients, contains eight questions, and can be administered by providers in about five minutes. The questions consider changes in food intake, weight loss, mobility, neuropsychological problems, body mass index, number of medications, patient’s perceived health compared to others of the same age, and age. The score categorizes patients as fit with a score of >14, intermediate risk with a score between 11-14, and vulnerable with a score of <14. In a large prospective study of 1435 cancer patients (ONCODAGE study), the G8 was found to have a sensitivity of 76.5% and a specificity of 64.4% of identifying patients who require GA compared to the reference of the multidimensional GA [25]. 

Specifically, for HNC patients, Gogineni et al. analyzed the G8 scores at baseline and after non-definitive treatment for 171 HNC patients undergoing stereotactic body radiation therapy (SBRT) with or without systemic therapy. Of the patients with a G8 score of <11 who were categorized as vulnerable, the median survival time was 13.2 months. Of the patients with a G8 score between 11-14 who were categorized as intermediate risk, the median survival time was 24.3 months. Of the patients with G8 scores >14 who were categorized as fit, the median survival time was 41.0 months. Patients with a decrease in their G8 score after treatment had lower survival (8.0 months) as compared to patients with increased or stable scores (36.0 months). Notably, the G8 scores did not correlate to any significant differences in the incidence of grade 3 or grade 4 toxicities [26]. These data suggest that the G8 score can be useful in categorizing approximate prognosis at baseline before radiotherapy and can also be useful in predicting prognosis after therapy. 

Bras et al. performed a prospective study on 259 patients with newly diagnosed HNC who were undergoing curative treatment and were assessed for frailty using a complete GA, GFI, and G8 score. Advanced tumor stage, major treatment intensity, concomitant CRT, and level of education were associated with acute RT-induced toxicity, but CGA, GFI, and G8 were only associated with postoperative outcomes, not RT toxicity [27]. Both studies suggest that the G8 correlates well with survival but does not have good efficacy for predicting RT-induced toxicity in HNC. 

Fernández-Camacho et al. performed a retrospective analysis to see if RT treatment modifications were made based on the results of the G8 and the CCI in 161 geriatric oncology patients [28]. They found that for the 28.7% of patients in their cohort that were vulnerable according to the G8, the radiotherapy was 5.8 times more likely to be modified. The treatment plans changed by up to 21% after analysis of frailty using the G8 [28]. Although the G8 score does evaluate frailty and vulnerability of patients with HNC undergoing treatment, it does not clearly evaluate or predict RT induced toxicity or its completion. 

SAOP2 

The SAOP2 is a tool used to determine which elderly patients with a cancer diagnosis should undergo more thorough multidisciplinary screening prior to treatment planning. This tool includes evaluation of independence, depression, self-rated health, ADLs, weight change, appetite, sleep quality, medication affordability, number of medications, and cognitive evaluation [29]. 

Russo et al. performed a prospective observational study with 282 patients over the age of 65 years with solid cancer diagnosis who were candidates for surgical, medical, or RT interventions. They compared the G8 and SAOP2 results against the gold standard of CGA, with each test being administered prior to treatment [30]. SAOP2 had 94% sensitivity for identifying patients who were also deemed frail based on the CGA, while the G8 had 89% sensitivity. The specificity for the SAOP2 was 46.9% [30]. These data suggest that the SAOP2 could be a good screening tool to identify patients who are at risk of increased frailty and poor treatment outcomes and warrant additional, more specific screening to guide treatment decisions. There are no studies looking at SAOP2 for GA of elderly patients undergoing HNC treatment, specifically RT. 

Short physical performance battery (SPPB) 

The SPPB is a test that assesses standing balance, gait speed, and the sit-to-stand test. These three components are totaled with a score of 12 indicating fully functional lower extremities and a score of <10 indicating increased risk of complications [31]. 

Farrugia et al. performed a prospective observational cohort study in 106 patients, with a mean age of 64, who received RT for HNC and evaluated functional decline using the SPPB [31]. Some patients underwent concurrent CRT, while a smaller proportion of patients received RT, with and without surgery. The SPPB was administered before and after their treatment. QOL was also assessed using questionnaires to evaluate a wide variety of functions. Frailty was assessed using the Fried Frailty Criteria. They found that a decline in SPPB after treatment correlated with a decline in quality of life and change in frailty status. This relationship was significant in the concurrent CRT group but not significant in the RT alone group. 

SPPB may be an underutilized tool that can be used to assess the patients most in need of rehabilitation during and after treatment. It has also been shown to be closely associated with other measures of frailty [31]. In a study (Wieland et al.) to identify the prevalence of the risk of oropharyngeal dysphagia, malnutrition, sarcopenia, and frailty and their co-occurrence in all newly diagnosed HNC patients, the risk of sarcopenia was measured using the SPPB and hand grip strength (HGS) [32]. HNC patients with a poor PS (2-3) had a significantly higher risk of sarcopenia (SPPB ≤ 9) compared to patients with a normal PS (0-1). However, HGS (<10th percentile), which is also a surrogate measurement for sarcopenia, was not significantly associated with a poor PS (2-3). SPPB provided information on physical disability and sarcopenia, with sarcopenia being a risk factor and physical disability being an outcome of frailty. There are some additional studies that have used SPPB to assess sarcopenia, weakness, muscle mass, functional status but not specifically RT-induced sarcopenia, functional decline from RT, or treatment completion [31,33,34]. 

eRFA 

The eRFA was developed to provide an effective and convenient electronic GA that can be done in the pre-operative setting for all geriatric patients in the field of surgical oncology. It assesses 12 domains, including functional status, cognition, social support, social activity interference, emotional status, nutrition status, vision, hearing, and polypharmacy. Based on each of these areas, an accumulated geriatric deficit (AGD) score is calculated, with a higher score correlating to a higher level of frailty. The test can be administered at home with the help of a caregiver or electronically in the office with the help of staff to complete the cognitive assessment and mobility testing [10]. 

Raab et al. performed a retrospective review of 159 patients over the age of 75 years who were planning to undergo HNC surgery [35]. Patients completed an eRFA 60 days prior to the surgery. They found that patients with higher AGD scores spent more time in the hospital post-operatively and had a higher risk of ICU admission compared to patients with lower AGD scores [35]. Since there were few deaths within 90 days of surgery, this study was not able to assess the effect of higher AGD scores on mortality. This tool is specific for patients undergoing surgical resection for HNC. 

CRASH 

The CRASH score was developed as the first score to predict the risks of severe chemotherapy toxicity for patients over 70 years of age. The scoring system contains a hematologic toxicity component and a non-hematologic toxicity component. The score includes the toxicity of the chemotherapy regimen. The hematologic component of the score includes diastolic blood pressure, an IADL score, and lactate dehydrogenase. The nonhematologic component includes ECOG PS, mental health status, and a nutritional assessment. The risks of each subcomponent can be measured, and an aggregate total risk score can be obtained [8]. 

Mittal et al. conducted a prospective observational cohort study with 100 patients over the age of 65 years who were receiving a new chemotherapy regimen, and their CRASH score was calculated [36]. Patients with the highest score categories for the hematologic, nonhematologic, and overall CRASH scores predicted nearly 100% risk of severe toxicity [36]. 

CARG-TT 

Like the CRASH score, the CARG-TT score was developed to predict severe toxicity in patients over 65 years of age. The score includes age, cancer type, dosage of chemotherapy, number of chemotherapy drugs, creatinine clearance, hearing, number of falls in the last six months, need for assistance taking medications, ability to walk one block, and social activity [9]. Mariano et al. conducted a prospective longitudinal study with 199 patients over 70 years of age receiving a new chemotherapy regimen [37]. The CARG-TT tool was administered before treatment, treatment was changed based on this score in five patients, and extra support was given to 38.5% of patients based on CARG-TT scores [37]. These data suggest that treatment decision-making can be altered based on the use of tools to decrease the rates of severe toxicity in chemotherapy administration for geriatric patients. 

Ortland et al. conducted an observational study with 120 patients over the age of 70 years who underwent chemotherapy and assessed their CRASH scores, CARG-TT scores, and toxicity [38]. They found that there was no significant difference between the predictive value of the CARG-TT score and combined CRASH score suggesting that both scores can be effective in predicting severe toxicity in geriatric patients undergoing chemotherapy [38]. 

Both CRASH and CARG-TT are designed for GA of patients undergoing chemotherapy. 

As shown above, there is no current GA tool that predicts RT toxicity or PT of RT treatment in HNC. 

GA tool for radiation therapy 

A GA tool for HNC patients undergoing RT or CRT, specifically to predict RT toxicity, should consider RT fields, dose, duration, primary site, and whether systemic treatment is being given (systemic or concurrent). This kind of GA tool for HNC patients undergoing RT can help us design the most appropriate treatment plan for all elderly patients. We have developed a GA for HNC patients undergoing RT treatment (unpublished) that considers RT treatment plan plus additional treatment factors to predict PT or extended treatment (ET) time. This tool was developed by head and neck radiation oncologists based on their clinical experience, judgment, and collaboration, as no currently available tool assesses RT-specific premature terminations. For each unit increase in total score of RT-GA, there was a significant increase in the risk of ET/PT (p = 0.002; odds ratio, 1.17; 95% CI: 1.06-1.28). On multivariable analysis considering these covariates, patients with an ECOG score of 2-4 had higher risk of ET/PT than patients with an ECOG score of 0-1 (p < 0.001; odds ratio, 3.63; 95% CI: 1.74-7.54). 

The list of GA tools has been listed in Table 1. 

**Table 1:** List of the GA tools that are used in solid oncology emphasizing HNC patients *, reference [39]; **, reference [40]; ***, reference [41] HNC, head and neck cancers; GA, geriatric assessment Author credits: Rebecca Epstein

Geriatric assessment tool	Main utility	Ease of use
CGA	Broad tool to evaluate functional limitations in many domains for geriatric patients	Very time-consuming and requires assessment from the multidisciplinary team
ECOG Performance Status	Assess functional ability and mobility	Fairly quick and assessed by MD or RN
VES-13	Screening tool to assess the increased risk of functional decline and death in the geriatric population	Self-reported by patients and quick
G8	8-question tool to assess risk specifically in geriatric cancer patients	Fairly quick and administered by MD or RN
SAOP2	Assess which elderly cancer patients should undergo further multidisciplinary evaluation to assess risk prior to treatment	Partially self-reported by patients and need MD or RN for part of the assessment
eRFA	Pre-operative tool used in surgical oncology to assess degree of frailty in geriatric patients	Self-reported by patients and quick
CRASH	Assess risk of severe chemotherapy toxicity in geriatric patients	Time-consuming and requires labs, vitals, and other assessments
CARG-TT	Assess risk of severe chemotherapy toxicity in geriatric patients	Administered by MD or RN and requires labs and other assessments
Cumulative Illness Rating Scale (CIRS-G)	Assess burden of comorbid disease in geriatric patients*	Assessed by MD or RN and based on thorough medical history
Geriatric Depression Scale	Self-reported depression screening tool for geriatric patients**	Fairly quick and self-reported by patient
Blessed Orientation Memory Concentration Test	6-question screening tool to assess cognitive impairment in geriatric patients***	Fairly quick and administered by MD or RN

## Conclusions

There are several tools that have been adapted or developed to quantify and assess geriatric risk in cancer treatment, such as the CGA, ECOG PS, G-8, VES-13, SAOP2, eRFA, SPPB, CRASH, and CARG-TT. Some of these tools are specific to geriatric patients but not cancer patients, some are specific to geriatric patients undergoing chemotherapy, and some are specific to surgical oncology. Although these tools are valuable for risk stratification of patients undergoing various cancer treatment modalities, none of the existing tools account for the unique risks associated with radiation therapy, particularly for HNC. We are currently prospectively validating a new geriatric risk assessment tool specific to radiation treatment that can accurately predict premature termination of treatment or ET time within our institutional HNC population. This tool will give radiation oncologists better accuracy when assessing risk and determining proper treatment modalities and radiation plans.
